# Risk Factors for Stoma Outlet Obstruction: Preventing This Complication after Construction of Diverting Ileostomy during Laparoscopic Colorectal Surgery

**DOI:** 10.31662/jmaj.2021-0187

**Published:** 2022-03-04

**Authors:** Kiyomitsu Kuwahara, Yasuji Mokuno, Hideo Matsubara, Masahito Uji, Ichiro Kobayashi, Shinsuke Iyomasa

**Affiliations:** 1 Department of Surgery, Yachiyo Hospital, Anjo, Japan

**Keywords:** laparoscopic colorectal surgery, diverting ileostomy, stoma outlet obstruction

## Abstract

**Introduction:**

Bowel obstruction at the outlet of the stoma, also referred to as “stoma outlet obstruction” (SOO), has been noted to be more common after laparoscopic colorectal surgery with diverting ileostomy than after laparotomy. Thus, the aim of this study is to identify the risk factors for SOO and to evaluate the effectiveness of a modified ileostomy procedure for reducing its incidence.

**Methods:**

The medical records of 63 patients who underwent laparoscopic colorectal surgery with diverting ileostomy between January 2014 and July 2021 were retrospectively reviewed. We analyzed the risk factors for SOO using computed tomography findings.

**Results:**

In total, 34 patients underwent surgery before modification of the ileostomy procedure (LSa group), and 29 patients underwent surgery after modification (LSb group). In the LSa group, 6 patients have reportedly developed SOO (SOO group), whereas 28 patients did not (non-SOO group). No patients in the LSb group developed SOO. The thickness of the abdominal rectus muscle (ThM) in the SOO group and the non-SOO group was 13.4 mm and 9.6 mm, respectively (*p* = 0.005). The angle between the ileostomy and the abdominal wall (AIW) was 95.8° in the non-SOO group and 82.2° in the SOO group (*p* = 0.033). The AIW was 93.4° in the LSa group and 99.7° in the LSb group (*p* = 0.043).

**Conclusions:**

As per our findings, a thick abdominal rectus muscle is predictive of SOO. Correction of the AIW (eliminating medial inclination) by modifying the operative technique has eliminated the occurrence of SOO in our patient population.

## Introduction

In recent years, diverting ileostomy has been identified as a common technique for colorectal surgery, especially when anal preservation surgery is desired, such as with ultralow anterior resection for lower rectal cancer, or when decreased incidence of anastomotic leakage is deemed necessary, such as with proctocolectomy for ulcerative colitis ^(1), (2)^. There are some reports describing the feasibility of diverting ileostomy for decreasing the incidence of anastomotic leakage and subsequent pelvic peritonitis ^(3), (4), (5), (6)^, and one report notes that fecal diversion can also lead to decreased morbidity and mortality associated with anastomotic leakage ^(7)^. However, a number of complications have been observed, and they are mostly associated with ileostomy, including dermatitis, stoma ischemia, parastomal hernia, and high-output syndrome ^(8)^.

Bowel obstruction at the outlet of the stoma called “stoma outlet obstruction” (SOO) is another complication of ileostomy, and a few reports note that SOO occurs more frequently with laparoscopic surgery than with laparotomy ^(9), (10)^. At our institution, several patients have reportedly experienced SOO after laparoscopic surgery; thus, we modified our operative technique for laparoscopic ileostomy construction on the assumption that pneumoperitoneum affects the incidence of SOO. The aims of this study are to identify the risk factors for developing SOO after ileostomy construction during laparoscopic colorectal surgery and to evaluate the effectiveness of a modified ileostomy procedure for reducing its incidence.

## Materials and Methods

We retrospectively reviewed the records of all patients who underwent laparoscopic colorectal surgery requiring construction of a diverting ileostomy between January 2014 and July 2021 at our institution. We excluded patients who did not undergo abdominal computed tomography (CT) between construction and closure of the ileostomy. This study was approved by the ethics board of Yachiyo Hospital, Japan (August 30, 2021). This study is retrospective observation research using medical data; therefore, we got informed consent by opt-out on the institutional website.

### Participants

We divided patients into two groups: those who underwent surgery before modification of the ileostomy procedure in April 2019 (the LSa group) and those who underwent surgery after modification of the procedure (the LSb group). We further divided the LSa group into those who did not develop SOO (the non-SOO group) and those who did develop SOO (the SOO group).

### Surgical technique for ileostomy

While pneumoperitoneum is still in place, we lift the skin of the abdomen using forceps and then make a circular incision (about 2.5 cm in diameter) at a site that was chosen and marked before pneumoperitoneum was established. We then dissect the subcutaneous tissues down to the fascia and make a cross-shaped incision in the anterior sheath of the rectus abdominis. We split the rectus muscle in the direction of its fibers until the posterior sheath is exposed; thereafter, we divide the posterior sheath, together with the peritoneum, and make the opening large enough to pass two fingers through. We pass the ileal loop through the opening and fix the serosa and muscular layer of the loop to the anterior sheath using 4 sutures. Proximal lumen of the ileostomy was orientated cephalad side in most patients not to be twist and suffer from intestinal tension. After deflation of pneumoperitoneum, we sterilize the surgical site and perform a transverse ileotomy. We then fix the everted wall of the incised ileum to the dermis using 8 sutures.

### Modification of the ileostomy procedure

Since April 2019, all procedures for creating the opening for ileostomy have been performed after deflation of pneumoperitoneum. We pass the ileal loop through the opening and then confirm that the loop is without tension.

### Definition of outcomes

**Figure 1.**
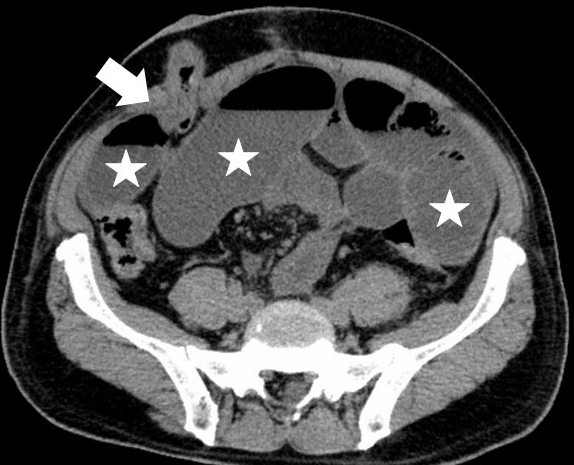
SOO patients were defined as those who had abdominal bloating symptoms and had CT findings of bowel obstruction at the stoma outlet and intestinal dilatation. White arrow head: bowel obstruction at the stoma, ☆: dilated intestines.

**Figure 2.**
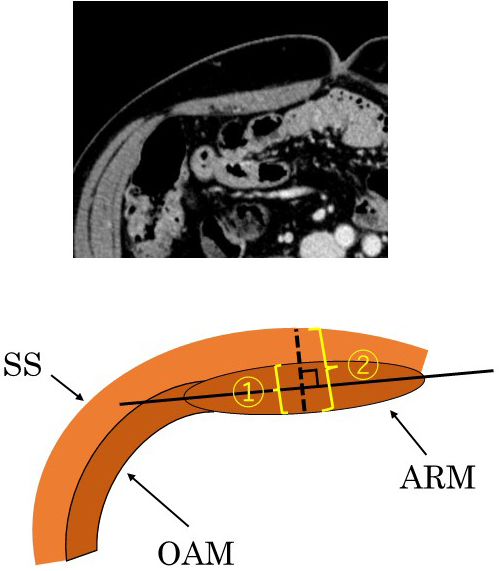
The thickness of the abdominal rectus muscle (①) is defined as the thickness of thickest part of the abdominal rectus muscle, orthogonal to the long axis of the abdominal rectus muscle. The thickness of the AIW (②) is defined as the thickness of the AIW along the same line. All measurements are made using an axial computed tomography view at the level of the umbilicus. SS, skin and subcutaneous; ARM, abdominal rectus muscle; OAM, external/internal oblique and abdominal transverse muscles.

**Figure 3.**
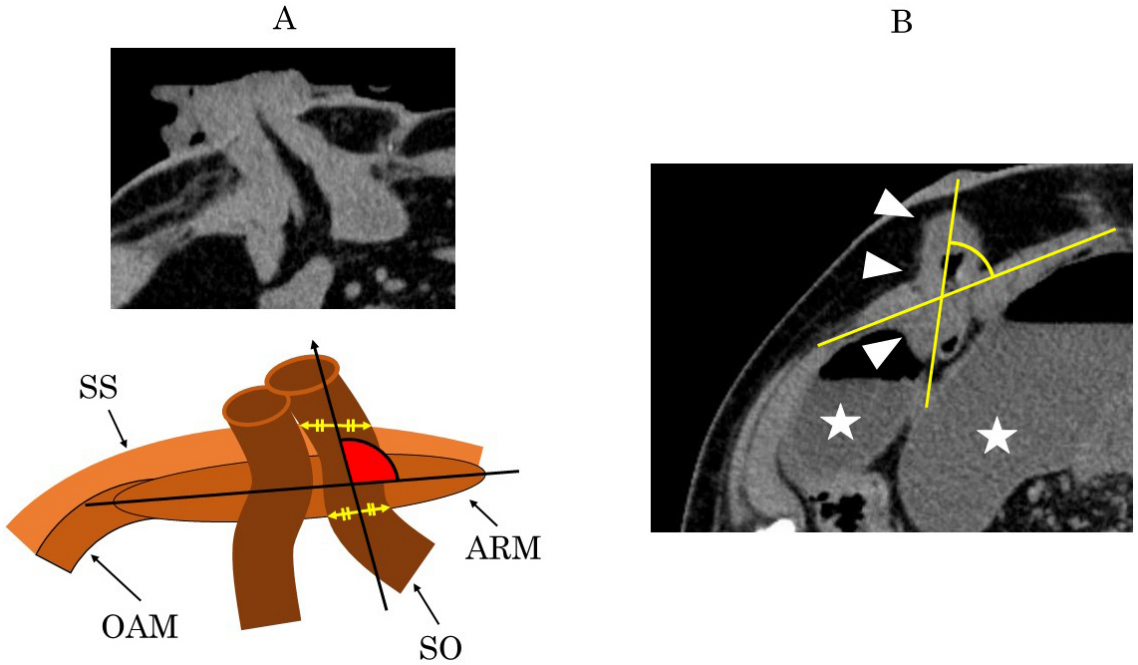
A. The angle between the ileostomy and the AIW is defined as the inner angle between the long axis of the abdominal rectus muscle and the straight line passing through two midpoints of the proximal lumen (at the skin and peritoneum level) (red area). All measurements are made using an axial computed tomography view. SS, skin and subcutaneous; ARM, abdominal rectus muscle; OAM, external/internal oblique and abdominal transverse muscles; SO, stoma outlet B. A CT for a patient who developed SOO. The CT findings show the proximal lumen inclines to the medial side. White arrow heads: stoma outlet, ☆: dilated intestines.

We defined SOO as a bowel obstruction occurring at the stoma outlet as it passes through the abdominal wall (AIW). Abdominal CT findings, clinical symptoms, and signs were used in order to confirm the diagnosis. SOO patients were defined as those who had abdominal bloating symptoms and had CT findings of bowel obstruction at the stoma outlet and intestinal dilatation (Figure 1). The outcomes of interest were colorectal disease, operative time, blood loss, thickness of the abdominal wall (ThW), thickness of the abdominal rectus muscle (ThM), the ratio of ThM to ThW, and the angle between the ileostomy and the abdominal wall. We then measured ThW and ThM using the axial view at the umbilical level on preoperative CT; further, we also measured AIW using the axial view on postoperative CT. The ThM was defined as the thickness of thickest part of the abdominal rectus muscle, orthogonal to the long axis of the muscle, whereas ThW was defined as the thickness of the AIW along the same line (Figure 2). The AIW was defined as the inner angle between the long axis of the abdominal rectus muscle and the midline of the proximal lumen of the ileostomy within the abdominal wall, as shown in Figure 3A. Figure 3B is a CT for a patient who had developed SOO. The CT findings show the proximal lumen inclines to the medial side.

We then compared results according to the occurrence of SOO and the surgical method employed.

### Statistical analysis

The data were expressed as mean ± standard deviation. Categorical variables were compared using chi-squared test or Fisher’s exact test. The Mann-Whitney U test or Student’s t-test were used to compare the continuous variables. A *p*-value of less than 0.05 was considered statistically significant. All statistical analyses were performed using the EZR program, version 1.41 (Saitama Medical Center, Jichi Medical University, Saitama, Japan).

## Results

In total, 67 patients underwent laparoscopic colorectal surgery with diverting ileostomy during the study period. Of these, 63 have met the inclusion criteria. In total, 34 patients underwent surgery before modification of the ileostomy procedure (the LSa group), while 29 patients underwent surgery after modification of the procedure (the LSb group). Six patients (17.6%) in the LSa group experienced SOO, while no patients in the LSb group suffered from this complication. The non-SOO subgroup of the LSa group had 28 patients.

### Risk factors for SOO

To assess the risk factors for SOO, we compared the SOO group with the non-SOO group. All were patients in the LSa group (n = 34; Table 1). Five patients with colorectal cancer and one with inflammatory bowel disease developed SOO. All patients were treated and cured with conservative treatment, and two patients required drainage for stoma outlet. No patient required surgery. No significant differences were observed in terms of sex, age, body mass index (BMI), blood loss, or operative time between groups. There was no apparent association between SOO and high-output syndrome that is stoma output more than 2000 ml/day. All patients who developed SOO were men.

**Table 1.** Comparison of the Characteristics of Patients Who Underwent Laparoscopic Surgery with Diverting Ileostomy between the Two Groups.

Variable	Non-SOO (n = 28) Mean ± SD or n	SOO (n = 6) Mean ± SD or n	*P*-value
Sex			0.297
Male	20	6	
Female	8	0	
Age (years)	67.1 (±10.0)	66.7 (±12.8)	0.944
Body mass index (kg/m^2^)	22.6 (±4.2)	22.8 (±1.6)	0.815
Colorectal disease
Colorectal cancer	24	5	
Inflammatory bowel disease	2	1	
Diverticular disease	2		
Blood loss (mL)	91.7 (±141.9)	122 (±100.0)	0.552
Operative time (min)	291.1 (±205.9)	301.1 (±141.2)	0.887
High output syndrome			0.126
2000ml or more/day	5	3
less than 2000ml/day	23	3
Thickness of			
Abdominal rectus muscle (mm)	9.6 (±1.8)	13.4 (±2.1)	0.005
Abdominal wall (mm)	28.8 (±9.2)	30.4 (±3.4)	0.476
The ratio of ThM/ThW	0.35 (±0.10)	0.45 (±0.09)	0.05
The angle of ileostomy (°)	95.8 (±9.1)	82.2 (±11.5)	0.033

The data are expressed as mean ±standard deviation (SD) or number only. ThM, thickness of the abdominal rectus muscle; ThW, thickness of the abdominal wall (Before the modification of ileostomy procedure: n = 34)

**Figure 4.**
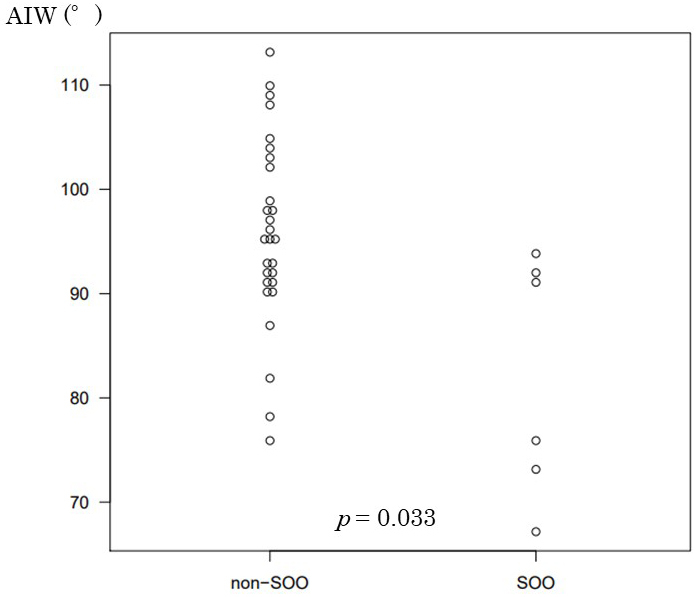
Comparison of the AIW between the stoma outlet obstruction (SOO) group and the non-SOO group. AIW, angle between the ileostomy and the abdominal wall; SOO, stoma outlet obstruction.

The ThM in the SOO group and the non-SOO group was 13.4 mm and 9.6 mm, respectively; this difference was deemed significant (*p* = 0.005). Although there was no significant difference for ThW, the ThM/ThW ratio was 0.35 in the non-SOO group and 0.45 in the SOO group; this difference was significant (*p* = 0.05). The AIW was 95.8° in the non-SOO group and 82.2° in the SOO group; this difference was significant (*p* = 0.033; Figure 4).

### Comparison of patient characteristics related to the stoma site and incidence of SOO between the LSa and LSb groups

**Figure 5.**
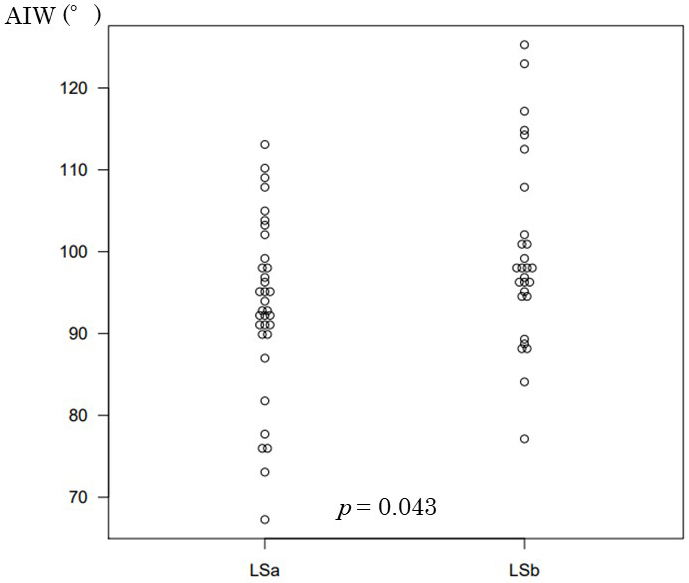
Comparison of the AIW between the group undergoing surgery before ileostomy modification (LSa) and the group undergoing surgery after ileostomy modification (LSb). AIW, angle between the ileostomy and the abdominal wall; LSa, group undergoing surgery before ileostomy modification; LSb, group undergoing surgery after ileostomy modification.

The patient characteristics related to the stoma site and incidence of SOO are shown in Table 2. No significant differences were noted in preoperative patient characteristics between the LSa and LSb groups, including the ThM and the ThM/ThW ratio. In total, six patients in the LSa group (17.6%) developed SOO; the AIW was 93.4° in this group. After we modified the surgical technique, the AIW was 99.7°, and it was no longer inclined to the medial side of the abdomen; this difference was significant (*p* = 0.043; Figure 5). No patients in the LSb group developed SOO.

**Table 2.** Comparison of The Patient Characteristics and Incidence of SOO between The LSa and LSb Groups.

Variable	LSa (n = 34) Mean ± SD or n	LSb (n = 29) Mean ± SD or n	*P*-value
Thickness of			
Abdominal rectus muscle (mm)	10.3 (±2.4)	9.2 (±2.7)	0.054
Abdominal wall (mm)	29.0 (±8.5)	29.1 (±10.9)	0.496
The ratio of ThM/ThW	0.37 (±0.10)	0.34 (±0.10)	0.091
The angle of ileostomy (°)	93.4 (±10.7)	99.7 (±12.3)	0.043
Incidence of SOO	6	0	0.027

The data are expressed as mean ±standard deviation (SD) or number only. SOO, stoma outlet obstruction; ThM, thickness of the abdominal rectus muscle; ThW, thickness of abdominal wall

## Discussion

This present study demonstrates three important factors related to the development of SOO after construction of a diverting ileostomy during laparoscopic colorectal surgery: a thick abdominal rectus muscle, a high ThM/ThW ratio, and an ileostomy inclined to the medial side. Correction of that angle with modification of the operative technique eliminated the occurrence of SOO in our study population.

Some studies have previously reported patient characteristics that are predictive of SOO ^(11), (12), (13), (14)^. Our findings show that a high ThM and a high ThM/ThW ratio are predictive factors for SOO. Okita et al. ^(15)^ report that a younger age (<16 years) at surgery and a low BMI (<21 kg/m^2^) may be significant predictive factors for SOO after construction of a diverting ileostomy in patients with ulcerative colitis. In contrast, Tamura et al. ^(16)^ report that SOO develops particularly in patients with obesity who have an especially thick subcutaneous fat layer in the AIW. Although the cause of these varying observations is unknown, a long pathway through the AIW may be the cause of SOO. Thus, we evaluated the condition of our ileostomies using postoperative abdominal CT and found that the angle of the stoma outlet lumen inclined to the medial side in patients with SOO. To the best of our knowledge, some studies have related the incidence of SOO to features of the abdominal rectus muscle ^(10), (13)^, but there are no reports describing the relation of morphologic ileostomy features to SOO.

In most patients, the proximal limb of the ileostomy runs from the medial abdomen to the right in a lateral direction; then, it turns and penetrates the AIW. We propose that this acute turn might be the cause of SOO. Reportedly, SOO occurs in 5.4% to 18.4% of patients undergoing laparoscopic ileostomy creation ^(10), (17), (18)^, more frequently than when the same procedure is performed via laparotomy ^(19)^. In our department, 6 of 34 patients with SOO had undergone laparoscopy, although we had never experienced SOO on laparotomy, during the study period. We, therefore, theorized that pneumoperitoneum affects the AIW and causes inclination of the ileostomy to the medial side of the abdomen. Based on this assumption, we modified our operative technique for ileostomy construction in April 2019 as described above. Consequently, the AIW in our patients has improved significantly, and we have had no patients experience SOO after the modification was implemented.

**Figure 6.**
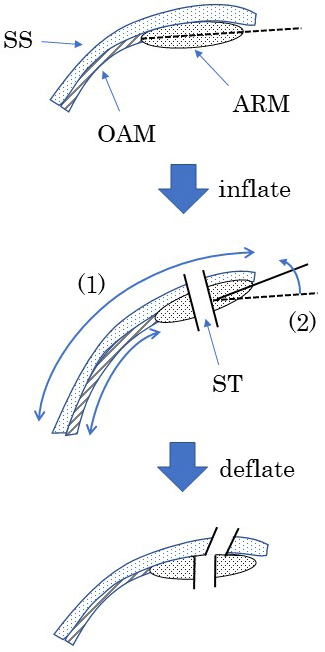
Two changes occur in the abdominal wall during pneumoperitoneum. (1) Due to the structural nature of the muscle fibers, the transverse abdominal muscle and the internal and external oblique muscles can extend laterally, but the abdominal rectus muscle cannot; thus, the skin and subcutaneous layer are displaced laterally from the muscular layer. (2) The angle between the long axis of the abdominal rectus muscle and the horizontal axis is increased. Due to these changes, the angle of the ileostomy outlet might tilt inward when the ileostomy is constructed under pneumoperitoneum. SS, skin and subcutaneous; ARM, abdominal rectus muscle; OAM, external/internal oblique and abdominal transverse muscle; ST, stoma trephine.

**Figure 7.**
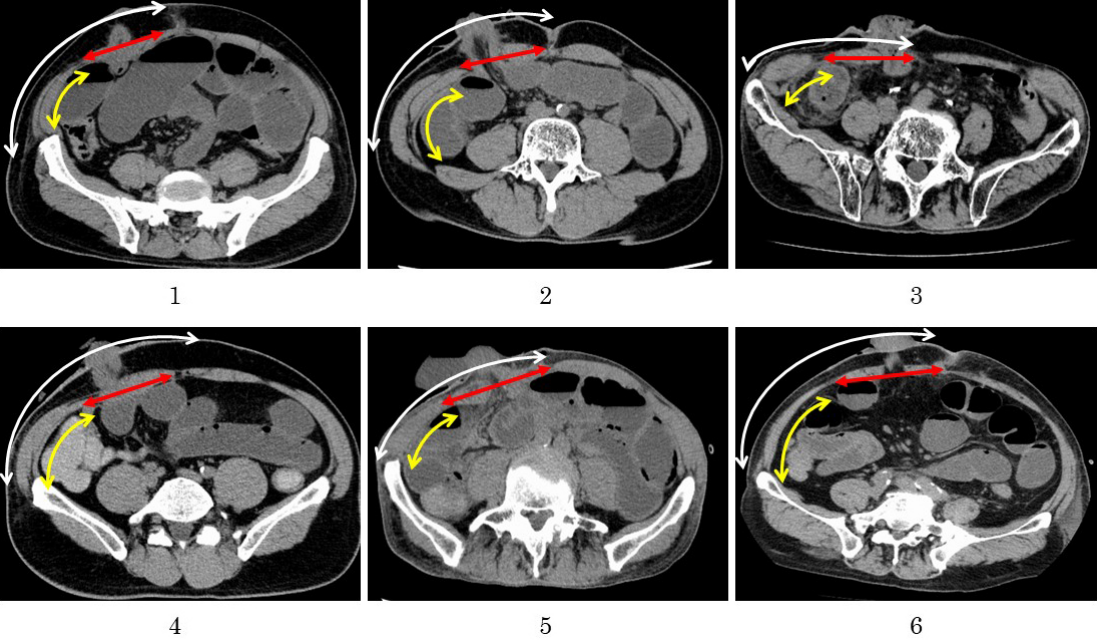
CT images of the six patients who have developed SOO. The transverse abdominal muscle and the internal and external oblique muscles extended laterally, but the abdominal rectus muscle did not under pneumoperitoneum. Thus, the skin and subcutaneous layer were displaced laterally from the muscular layer. Yellow arrow: transverse abdominal muscle and internal and external oblique muscles, red arrow: abdominal rectus muscle, white arrow: skin and subcutaneous layer.

We postulate that pneumoperitoneum creates two changes in the AIW (Figure 6). First, the transverse abdominal muscle and the internal and external oblique muscles can extend laterally, due to the structural nature of their muscle fibers, but the abdominal rectus muscle cannot. Thus, the skin and subcutaneous layer are displaced laterally from the muscular layer during pneumoperitoneum (Figure 7). Second, the angle between the long axis of the abdominal rectus muscle and the horizontal axis is increased via pneumoperitoneum. Due to these changes, the angle of the ileostomy outlet might tilt inward when the ileostomy is constructed under pneumoperitoneum. The fact that ThM correlates with the incidence of SOO is explained by the theory that these changes might be particularly apparent when the abdominal rectus muscle is thick.

A few limitations associated with this study warrant mention. For one, this is a retrospective study with a small number of patients, conducted at a single institution. To evaluate the true risk factors for SOO and to determine the appropriate prophylactic measures, a prospective study with a large number of patients is thus needed. Additionally, surgeons were not blinded to the difference in characteristics between the LSa group and the LSb group. All surgeons knew that the AIW was at an acute angle in patients with SOO. Thus, it is possible that they used techniques other than pneumoperitoneum deflation to eliminate the inclination of the ileostomy in the LSb group. Accordingly, it remains unclear how much the pneumoperitoneum deflation affected the decreased incidence of SOO. Despite these limitations, we are convinced that our analysis was able to demonstrate that an acute AIW is strongly related to the occurrence of SOO and that modifying the operative procedure results in eliminating SOO.

We conclude that a thick abdominal rectus muscle is a predictive risk factor of SOO and a medially inclined AIW after ileostomy for colorectal surgery is a surgical risk factor. Both risk factors are important causes of SOO. In operation that changes in the abdominal wall under pneumoperitoneum may result in encouraging this inclination. Correction of the AIW by modifying the operative procedure, including deflating the pneumoperitoneum before ostomy creation, has eliminated the occurrence of SOO in our patient population. We expect these findings will contribute to a wide decrease in the development of SOO after laparoscopic colorectal surgery.

## Article Information

### Conflicts of Interest

None

### Author Contributions

All authors listed in the manuscript meet the ICMJE contribution criteria.

### Approval by Institutional Review Board (IRB)

This study was approved by the ethics board of Yachiyo Hospital, Japan (August 30, 2021). We don’t have approval code in our hospital.
